# Microbiota and Metabolite Profiling Combined With Integrative Analysis for Differentiating Cheeses of Varying Ripening Ages

**DOI:** 10.3389/fmicb.2020.592060

**Published:** 2020-11-26

**Authors:** Roya Afshari, Christopher J. Pillidge, Daniel A. Dias, A. Mark Osborn, Harsharn Gill

**Affiliations:** ^1^School of Science, RMIT University, Bundoora, VIC, Australia; ^2^School of Health and Biomedical Sciences, RMIT University, Bundoora, VIC, Australia

**Keywords:** cheese, 16S rRNA-based microbiota analysis, GC-MS untargeted metabolomics, cheese maturity, integrative analysis

## Abstract

Cheese maturation and flavor development results from complex interactions between milk substrates, cheese microbiota and their metabolites. In this study, bacterial 16S rRNA-gene sequencing, untargeted metabolomics (gas chromatography-mass spectrometry) and data integration analyses were used to characterize and differentiate commercial Cheddar cheeses of varying maturity made by the same and different manufacturers. Microbiota and metabolite compositions varied between cheeses of different ages and brands, and could be used to distinguish the cheeses. Individual amino acids and carboxylic acids were positively correlated with the ripening age for some brands. Integration and Random Forest analyses revealed numerous associations between specific bacteria and metabolites including a previously undescribed positive correlation between *Thermus* and phenylalanine and a negative correlation between Streptococcus and cholesterol. Together these results suggest that multi-omics analyses has the potential to be used for better understanding the relationships between cheese microbiota and metabolites during ripening and for discovering biomarkers for validating cheese age and brand authenticity.

## Introduction

In large-scale cheddar cheese manufacture, starter bacteria (normally selected strains of *Lactococcus lactis*) together with adjunct bacteria (these may or may not be added and typically comprise strains of *Lactobacillus* spp. and/or other bacteria) are inoculated into the milk (Fox et al., 2017). As these bacteria grow, they produce lactic acid and break down milk proteins (caseins) to release peptides and amino acids and also produce many diverse secondary metabolites that determine the final quality and flavor of cheese (Fox et al., 2017). Cheese ripening is a highly complex and time-dependent process that is necessary for full flavor development. Ripening involves successional changes in microbial communities and in their associated enzymatic and biochemical reactions that underpin the release of hundreds or thousands of flavorsome compounds (Blaya et al., 2018). While it has long been established that balanced ripening is pivotal for optimum quality and flavor development (Ochi et al., 2013), being able to consistently predict and control cheese maturation processes between batches during cheesemaking remains a challenge, even for large-scale commercial operations. Following production, cheeses are graded for quality and those of lower quality are commonly diverted into processed cheese manufacture, but at a reduced price. In recent years, DNA sequencing has revealed that in addition to lactic acid bacteria (LAB), other adventitious species may also be present during ripening with their associated role in cheese ripening less well-understood. These adventitious species or taxa include *Arthrobacter* spp., *Prevotella*, *Faecalibacterium* (Quigley et al., 2012), marine-associated γ-Proteobacteria (Wolfe et al., 2014), coagulase-negative staphylococci, members of the *Enterobacteriaceae* and other unclassified genera (Yeluri Jonnala et al., 2018).

The application of high-throughput DNA sequencing and metabolomics approaches combined with new computational algorithms and data-analysis platforms (multi-omics) are now providing significant advances in understanding of the complex microbial and metabolic interactions involved in cheese ripening (Walsh et al., 2020; Afshari et al., 2018; Wolfe et al., 2014). Together, they have the potential to provide important new understanding of cheese production and maturation processes and the potential for identification and application of biomarkers that could be used to predict, optimize and control cheese ripening outcomes (Afshari et al., 2018). This is important as the global consumption of cheese is projected to increase by ∼13% between 2016 and 2025 (OECD/FAO, 2016). Consumers are increasingly demanding high-quality products with excellent sensory properties at a reasonable cost (Braghieri et al., 2014). In response, large cheese manufacturers typically map consumer preferences in different geographical and demographic markets, aiming for optimally targeted products within competitive sales environments, for example as has been explored for Cheddar cheeses of different maturities (Young et al., 2004). The provision of improved tools to discriminate between cheese of varying quality that could be incorporated into cheese manufacturing processes during cheese ripening would aid manufacturers so that their final products can increasingly and more consistently closely match the preferences of consumers.

This research has applied a multi-omics approach combining 16S rRNA-based microbiota and untargeted metabolomics [gas chromatography-mass spectrometry (GC-MS)] analyses in combination with data integration analysis to investigate the interrelationships between cheese microbiota and metabolomes in Cheddar cheeses from different manufacturers and of varying maturity (ripening age). The aims of this research were to identify key microbiota and/or metabolites that are characteristic of these cheeses and to determine interrelationships between these microbiota and metabolites.

## Materials and Methods

### Sampling

Cheddar cheeses produced by three Australian commercial manufacturers were purchased from local supermarkets (these three brands of cheddar were designated in this study as A, B, and C). For each brand, cheeses of three or four different maturities were available from each manufacturer. For brand A, four types of cheese were purchased: “mild,” “tasty,” “extra tasty,” and “vintage.” However, no specific ripening times were stated on the packs. For Brand B, cheese was labeled as “tasty” (ripened up to 12 months), “extra-tasty (ripened for up to 18 months)” or “epicure” (ripened for up to 32 months). For brand C, each cheese was labeled as “sharp” (ripened for up to 12 months), “extra-sharp” (ripened for up to 20 months) or “special reserve” (ripened for up to 32 months). For simplicity, in this study we have labeled cheeses from all three brands using the same terminology (in increasing order from minimum to maximum ripening level). These definitions are “mild” (up to 6 months), “tasty” (up to 12 months), ‘extra-tasty’ (up to 18 months) and ‘vintage’ (up to 32 months). We have also assumed that the ripening times for the four brand A cheeses are approximately similar to those of the corresponding cheeses for brands B and C. For each brand and level of ripening, four 250 g (approximate) commercially packaged shrink-wrapped blocks were purchased and sampled aseptically. Each individual sample was divided into two sub-samples; one of these was frozen at −80 °C until subsequent DNA-sequencing analysis, while the second was immediately homogenized using a mortar and pestle with liquid nitrogen and then freeze-dried for subsequent GC-MS metabolomics analysis.

### 16S rRNA-Based Cheese Microbiota Analysis

Total DNA was extracted from 200 mg of each cheese sample using a PowerSoil DNA Isolation Kit (MO BIO Laboratories, Inc., Carlsbad, CA, United States) following the manufacturer’s instructions. DNA purity and concentration were determined and PCR amplification and DNA sequencing of bacterial 16S rRNA genes were performed as described previously (Afshari et al., 2020). Briefly, the V4 region of DNA was amplified using primers 515F and 806R. PCR conditions consisted of 95°C for 3 min, followed by 25 cycles of: 95°C for 30 s, 55°C for 30 s, 72°C for 30 s and a final extension at 72°C for 5 min. 16S rRNA amplicons were purified and indexed using the Nextera XT DNA library prep kit as according to the 16S Metagenomic Sequencing Library Preparation instructions (Illumina, San Diego, CA, United States). Indexed PCR amplicons were pooled in equal concentrations and sequenced on an Illumina MiSeq platform (Illumina, San Diego, CA, United States).

Raw Illumina fastq files were demultiplexed, quality filtered, and analyzed using GHAP v2.1 (Greenfield Hybrid Amplicon Pipeline, developed by Paul Greenfield at CSIRO, Canberra, ACT, Australia) as described previously (Afshari et al., 2020). The relative abundance of each taxon in each sample was determined using the vegan package Rv.3.4.3. Beta diversity was calculated based on a Bray-Curtis dissimilarity matrix using Primer v7 (Primer-E, Plymouth, United Kingdom). A non-metric-multi-dimensional scaling (nMDS) plot was generated from the resulting distance matrix. Permutational multivariate analysis of variance (PERMENOVA) with 999 permutations was used to test the significant differences in phylogenetic diversity between cheeses of different ages within and between each manufacturer based on a Bray-Curtis matrix. Since the number of unique permutations was less than 50, the marginal *p*-value which was significant in PERMANOVA was further tested by Monte Carlo analysis.

### Gas Chromatography-Mass Spectrometry Metabolomics

Freeze-dried cheese (60 mg) was extracted as described by Afshari et al. (2020). Briefly, 60 mg of cheese was extracted in 500 μL of MeOH/H_2_O/CHCl_3_ (2.5:1:1, *v*:*v*:*v*). Internal standards (100 μL of ^13^C_6_-sorbitol/^13^C_5_^15^N-valine in water, 0.2 mg mL^–1^) were then added to this mixture. The extract was homogenized using a MP homogeniser (FastPrep)^®^ for 1 min at 4.5 m/s, then incubated at 37°C for 15 min in a thermomixer at 850 rpm, centrifuged at 15700 *g* for 15 min and the supernatant was then decanted into a new tube. The remaining pellet was mixed with 500 μL of MeOH/H_2_O/CHCl_3,_ centrifuged at 13000 rpm for 15 min and the resulting supernatant was then combined with the previous extract. Following extraction, 40 μL aliquots were transferred into glass vial inserts and dried *in vacuo* for subsequent trimethylsilyl (TMS) polar metabolite derivatization. Online, chemical derivatization and acquisition were performed as previously described by Afshari et al. (2020). This method specifically enables the extraction of non-volatile compounds.

Resulting GC-MS data were analyzed using the Agilent Mass Hunter Workstation software, Quantitative analysis, Version B.07.01/Build 7.1.524.0 (Agilent Technology, Inc). Mass spectra of eluted compounds were identified using the commercial mass spectra library NIST 08,^1^ the public domain mass spectra library of Max-Planck-Institute for Plant Physiology, Golm, Germany,^2^ an *in-house* mass spectral library at RMIT University and also by comparing their retention time to authentic standards. Relative response ratios (area of analyte divided by area of the internal ^13^C_6_-sorbitol standard and sample dry weight) were calculated for each analyzed metabolite and used for multivariate analysis. Principal component analysis (PCA) was used to analyze the GC-MS data of cheeses between and within each manufacturer. Principal component analysis and PCA biplots were performed in SIMCA 15.0.1 (Umetrics AB, Umea, Sweden).

### Data Integration Analysis

Multifactorial analyses (MFA) were performed in R using the FactoMineR package^3^ to assess variation with respect to cheese maturity based on the microbiota and metabolite compositions of cheeses and to find canonical correlation between metabolite and microbiota profiles. Multifactorial analysis is a generalization of PCA in which sample similarity is determined by multiple different sets of variables (Escofier and Pages, 1994), in this case, microbiota and metabolite profiles.

Random forest (RF) is a non-parametric machine learning technique, where multiple regression or classification trees are constructed using RF subsets of the data (Breiman, 2001). While a linear regression would fit only a linear relationship between the predictors and the outcome, RFs allow for any type of relationship, including complex interactions. Random forest analysis was used to predict associations between taxa and metabolites (regression model) for each manufacturer. Over 500 trees were constructed using the RF package and a 10-fold cross-validation was used to evaluate these RFs. Based on the mean decrease in Gini-coefficient, the most ‘important’ parameters were selected (Louppe et al., 2013). The variable with the highest mean decrease in Gini index is considered the most important variable in the optimized model. Random forest analysis does not provide a regression coefficient; therefore, partial plots were used to show the adjusted relationship between the taxa and metabolites as other metabolites are held constant at their mean observed value (Friedman, 2001). The PartialPlot function in R was used to generate partial dependence plots for the five most important variables.

## Results

### Bacterial Community Structure in Cheddar Cheeses of Different Brands and Age

**FIGURE 1:**
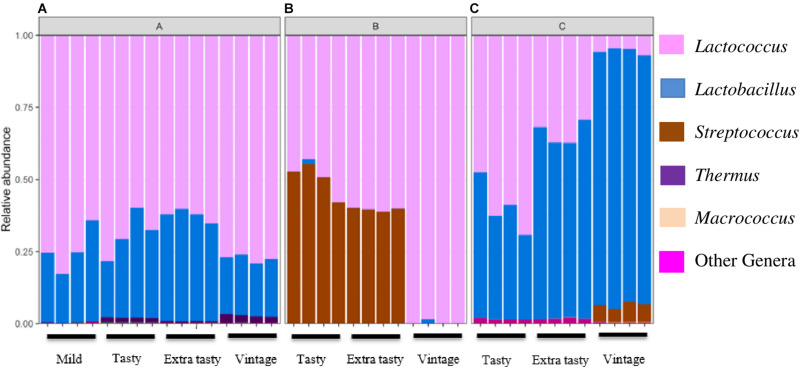
Variation in the relative abundance of bacterial genera in cheeses between brands and between cheeses of different maturity. Brands **(A–C)** are as indicated. Only genera detected at >0.1% relative abundance are shown.

Sequencing of PCR-amplified 16S rRNA gene amplicons was applied to investigate variation in the bacterial communities in cheddar cheeses of different brands and ages. Across all samples, a total of 115 operational taxonomic units (OTU) were identified at 97% identity. At the phylum level, Firmicutes comprised the highest proportion of detected OTUs (84% of all OTUs) and more than 99% of all sequence reads (Figure 1). Twelve genera dominated across the cheeses. For brand A, *Lactococcus* and then *Lactobacillus* were most dominant (Figure 1A). For brand B, *Lactococcus* and *Streptococcus* were most abundant in tasty (up to 12-month ripened) and extra-tasty (up to 24-month ripened) cheeses, while *Lactococcus* (only) was dominant in vintage cheeses (up to 32-month ripened) (Figure 1B). For brand C, the bacterial community was dominated by *Lactococcus* and *Lactobacillus* in varying proportions in tasty and extra tasty cheeses with *Lactobacillus* present in higher proportions (over 90%) in vintage cheeses (Figure 1C). Within these cheeses *Streptococcus* (presumably mostly *Streptococcus thermophilus*) and *Macrococcus* (0.1% of total reads) were also present at low abundance, except for brand B where *Streptococcu*s constituted up to more than 50% of the communities in the tasty and extra tasty cheese (Figure 1B). *Thermus* was present at low abundance in all brand A cheeses with a maximum of 2.5% of total reads in Brand A vintage cheeses (Figure 1A).

**FIGURE 2:**
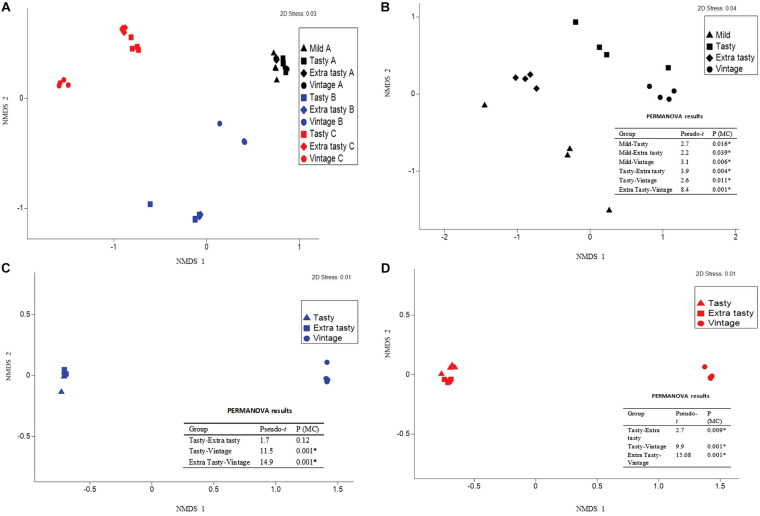
Variation in bacterial community composition in cheese between brands and between cheeses of different maturity. Bray-Curtis non-metric multi-dimensional scaling (nMDS) plots and PERMANOVA results are shown comparing **(A)** variation between cheeses from different manufacturers and **(B–D)** variation between cheeses of different maturity for each manufacturer: brand A **(B)**, brand B **(C)**, and brand C **(D)**. Different colors and shapes show different cheese manufactures and different ages, as indicated. PERMANOVA results are shown; Pseudo-*t* = the Pseudo-*t* statistic, whereby higher values indicate greater differences between the community structure of cheeses of the two groups compared; P (MC) = Monte Carlo *P-value*, with significant values (*P* < 0.05) indicated by an asterisk.

The bacterial community composition in the cheeses differed more between brands than within brands (Figure 2A), with permutational multivariate analysis of variance confirming that this variation between brands was statistically significant (*P* < 0.001). Within each brand, the bacterial communities within cheeses of different maturities all varied significantly from each other (*P* < 0.05; Figures 2B–D) but with the exception of the bacterial communities in tasty and extra tasty cheeses from brand B in which there was no significant variation between these communities (*P* = 0.12; Figure 2C).

### Variation in Metabolome Profiles in Cheddar Cheeses of Different Brands and Ripening Age

**FIGURE 3:**
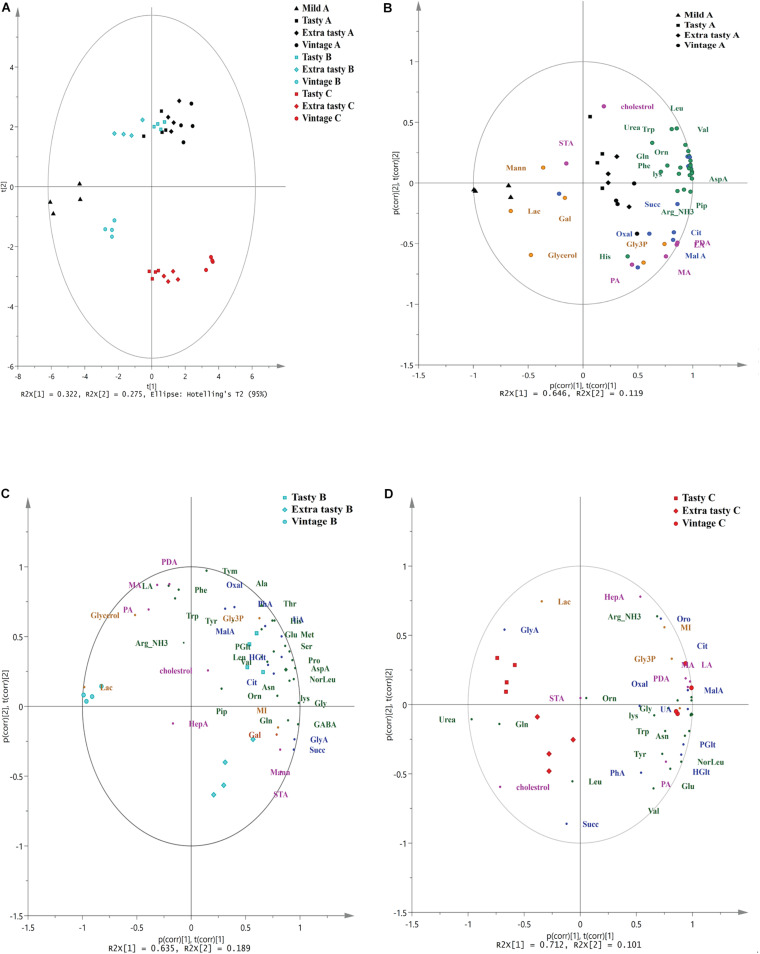
Principal Component Analysis (PCA) of untargeted GC/MS metabolomics of cheeses made by different manufacturers. **(A)** PCA of untargeted GC/MS metabolomics of cheeses made by different manufacturers. PC1 and PC2 account for 32.2% and 27.5% of the variance, respectively. **(B–D)** The biplot superimposed on the scores and loadings of PCA analysis based on a correlation scaling method for cheeses for brand A **(B)**, brand B **(C)**, and brand C **(D)** Brands and maturity of cheeses are as indicated. p(corr), t(corr) is a combined vector, p(corr) represents loading p scaled as correlation coefficient between X and t; t(corr) represents score t scaled as correlation coefficient resulting in all points falling inside the circle with radius 1. Different colors represent different brands and different classes of metabolites: black; brand A, cyan; brand B, red; brand C, green; amino acids and amines, blue; carboxylic acids, pink; fatty acids and sterols, orange; sugar and sugar phosphates. Orn, ornithine; Tyr, tyrosine; GABA, gamma amino butyric acid; Lys, lysine; Gly, glycine; Val, valine; Ser: serine; Leu, leucine; Noreleu, noreleucine; Thr, threonine; Pro, proline; Pip, piperedine; Asn, asparagine; AspA, aspartic acid; Glu, glutamic acid; Met, methionine; Arg, arginine; PDA, pentadecanoic acid; HepA, heptadecanoic acid; LA, lauric acid (dodecanoic acid); PA, palmitic acid (hexadecanoic acid); STA, stearic acid (octadecanoic acid); MA, myristic acid (tetradecanoic acid); Oxal, oxalic acid; Succ, succinic acid; GlyA, glyceric acid; Glt, glutaric acid; MalA, malonic acid; HGlt, hydroxy-glutaric acid; Citric, citric acid; GalA, galactonic acid, Pglu, pyroglutamic acid; Oro, orotic acid; UA, uric acid; PhA, phosphoric acid; Mann, mannose; Gal, galactose; MI, inositol myo; Lac, lactose; Gly3p, glycerol-3-phosphate.

Gas chromatography-mass spectrometry untargeted metabolomics profiling revealed a total of 46 primary metabolites across all cheese brands and maturities. These metabolites comprised of 22 amino acids and amines, 11 carboxylic acids, seven free fatty acids and steroids and six-sugar and -sugar derivatives (Supplementary Table 1). Principal component analysis showed that the variance between cheese samples based on their metabolite profiles (Figure 3). The first two principal components accounted for over 59% of the total variance (32.5 and 26.8% for PC1 and PC2, respectively) in the metabolome data. PCA showed that the metabolite profiles of brand C cheeses clustered separately from those of brands A and B (Figure 3A). Within brands, metabolite profiles also varied between cheeses of different maturities. Within brand A, metabolite profiles of mild cheeses (shown as triangles) were distinct from those of the more mature cheeses. Conversely, within brand B and C cheeses, metabolite profiles of vintage cheeses (circles) were distinct from those in tasty and extra-tasty cheeses. For all three brands, PC1 was the component which explained the largest proportion of the variance and best characterized the level of cheese maturity. To further investigate the relationships between individual metabolites and cheese maturity, PCA biplots were generated (Figures 3B–D).

The PCA biplot of brand A cheeses (Figure 3B) showed that mild cheeses contained a higher relative abundance of glycerol, lactose and mannose, whereas the mature cheeses (extra tasty and vintage) had higher relative abundances of numerous amino acids and carboxylic (such as citrate, malate, oxalate, succinate, and hydroxy-glutaric acid), free fatty acids (such as pentadecanoic acid, myristic acid, lauric acid, and palmitic acid) and also one amine (piperidine). This might be expected as the number of such metabolites would increase as ripening progresses. In contrast and perhaps surprisingly, in brand B cheeses (Figure 3C), these metabolites were present in higher relative abundance in the tasty cheeses when compared to the more mature extra tasty and vintage cheeses. More specifically, a total of 16 amino acids, and four carboxylic acids (citrate, oxalate, orotic, and uric acids) were more strongly associated with the brand B tasty cheeses. In contrast, only seven metabolites (GABA, glutamine, succinic acid, glycerate, octadecanoic acid, inositol, and galactose) were found to be present in higher abundance in the extra tasty cheeses while vintage cheeses were highly associated with just two metabolites, urea and lactose (Figure 3C). For brand C (Figure 3D), the relative abundance of 19 amino acids and one amine (piperidine) were strongly associated with the vintage cheeses, whereas (in contrast) leucine was more strongly associated with the extra tasty cheeses and glutamine with both tasty and extra tasty cheeses (Figure 3D). The relative abundance of carboxylic acids and fatty acids were also higher in the brand C vintage cheeses with the exceptions of succinic acid which was more strongly associated with the extra tasty cheese and of stearic acid which was present in similar proportions in all brand C cheeses.

Overall, GC-MS untargeted metabolomics profiling showed that there was an increase in the relative abundance of amino acids and amines, carboxylic acids and free fatty acids (especially malic acid, hydroxy-glutaric acid, citric acid, lauric acid, myristic acid pentadecanoic acid and palmitic acid) for brands A and C which correlated positively with increasing cheese age (PC1). For brand B this increase unexpectedly ceased after the cheese had aged beyond 12 months (i.e., tasty).

### Determining Relationships Between Cheese Microbial Composition, Metabolome Profiles, and Cheese Maturity

**FIGURE 4:**
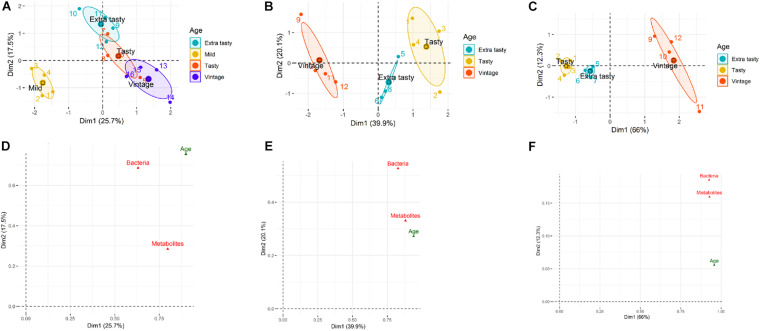
Multifactorial analysis (MFA) of cheese microbiota and cheese metabolite profiles for cheeses of different maturities from different manufacturers (A, B, and C). **(A–C)** MFA scatter plots for cheeses from brands A, B, and C, respectively. Ellipses representing the barycentre of the sample groups with 95% confidence. Maturity (age) of chesses is as indicated. **(D–F)** MFA group representations to illustrate the relationships between variables (bacterial genus composition, metabolite profiles, maturity of cheeses) and Dim 1and Dim 2 for brands A, B, and C, respectively.

Multifactorial analysis was used to determine the similarity/dissimilarity between cheeses of different ages within each brand based on the combined microbiota and metabolites profiles (integrated multi-omics datasets). Multifactorial analysis was also used to investigate relationships between cheese microbiota, cheese metabolites and cheese age within each brand and to identify correlations between individual bacterial taxa and metabolites. The scatter plots visualized the cheeses into a two-dimension space using the first two dimensions (Dims) which captured 43.2, 60, and 88.3% of the total variability among cheeses within brand A, B, and C, respectively (Figures 4A–C). The scatter plots showed that cheeses of different ages made by the same manufacturer could be separated based on the combined microbiota (bacterial taxa) and metabolite profiles (Figures 4A–C). Figures 4D–F identifies correlations between variables (herein: microbiota, metabolites, and age) and dimensions of MFA scatter plots for each brand. For brand A, the coordinate of metabolites on Dim 1 is higher than for microbiota, indicating the greater contribution of metabolites compared to the microbiota to the separation of mild cheeses from the other more matured cheeses (Figure 4D). For brand B, the contribution of microbiota and metabolites to Dim 1 is almost identical. However, on Dim 2 for which tasty and extra tasty cheeses were separated from each other, the contribution of microbiota was higher (Figure 4E). For brand C cheeses, both microbiota and metabolites had identical contributions to Dim 1 and very similar contributions for Dim 2 (Figure 4F). This could reflect the higher correlation that was obtained between these two datasets (microbiota and metabolites) (RV = 0.73) for cheeses of brand C when compared to other two brands (for brand A, RV = 0.33; for brand B, RV = 0.57).

**FIGURE 5:**
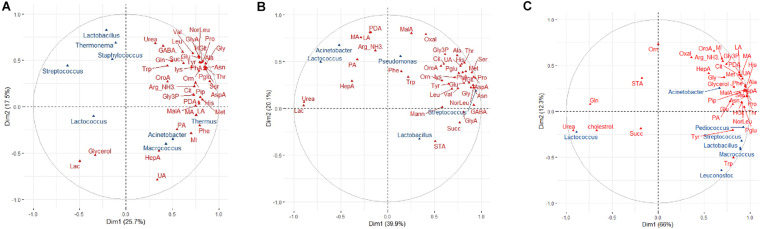
Multifactorial analysis (MFA) showing correlations between bacterial genera and metabolites in cheese. The Correlation circle depicts correlations in normalized abundance between cheese, bacterial genera (blue) and cheese metabolites (red) along the MFA axes for **(A)** brand A, **(B)** brand B, and **(C)** brand C cheeses. Those metabolites and genera which are depicted together in the same direction along an axis indicate positive correlations; those which are depicted together in the opposite direction indicate negative correlations; metabolites and genera depicted in different directions indicate no correlations. The strength of the correlation is shown by the increasing distance from the center of the plot. To improve the readability of the plots, only the greatest correlations for metabolites in each dimension are shown.

Numerous significant correlations were observed between specific bacterial taxa and metabolites for brands B and C (Figures 5B,C). For brand A cheeses, however, this correlation was less pronounced (RV coefficient = 0.33) (Figure 5A). The only notable exception was the positive correlation between *Thermus* and relative abundance of phenylalanine (Figure 5A). The relationship between age and metabolites for brand A (RV coefficient = 0.65) was also stronger than the relationship between age and microbiota profiles (RV coefficient = 0.57). Similarly, for brands B and C cheeses, the relationships between age and metabolites (RV coefficients = 0.81 and 0.77, respectively) were stronger than the relationships between age and microbiota (RV = 0.50 and 0.58, respectively). The MFA correlation circle for brand B and specifically for brand C revealed several positive microbiota-metabolite relationships. These included, for brand B, relationships between both *Lactococcus* and *Acinetobacter* (present at low abundance) and medium chain fatty acids including lauric acid, myristic acid, pentadecanoic acid and palmitic acid; and between *Streptococcus* and the relative abundance of amino acids (Figure 5B). For brand C, relationships were found between both *Streptococcus* and *Pediococcus* (but not *Lactococcus*) and increased relative abundances of amino acids which have sensory properties, including pyroglutamate, tyrosine, and proline (Figure 5C). In addition, *Streptococcus* was found to be associated with decreased abundance of both cholesterol and urea in brand C.

### Random Forest Analysis to Predict Associations Between Cheese Metabolites and Bacterial Genera

Random forest regression analysis was used to predict the association between the dominant bacterial genera and the cheese metabolites within each brand. For brand A cheeses, the optimized model showed that the relative abundance of *Thermus* was highly positively associated with phenylalanine (pseudo *R*^2^ = 0.85; Table 1 and Supplementary Figure 1A), supporting the results of MFA. The RF model did not identify any significant association between the microbiota and other metabolites for brand A. For brand B, the optimized model showed that the relative abundance of *Streptococcus* was negatively associated with the levels of urea and lactose (pseudo *R*^2^ = 0.93; Table 1 and Supplementary Figure 1B) and conversely that *Lactococcus* (presumably *Lc. lactis* added as the starter culture) was positively associated with heptadecanoic acid (Table 1 and Supplementary Figure 1C). For brand C (Table 1 and Supplementary Figures 1D–F), *Streptococcus* was associated with decreased cholesterol and urea, whilst *Lactococcus* was associated with decreased pyroglutamic acid and piperidine levels, and increased urea (pseudo *R*^2^ = 0.96). *Lactobacillus* in brand C cheeses was associated with decreased ornithine (not present in casein) and glutamine levels and increased tyrosine.

**TABLE 1:** Adjusted associations between the bacterial genera and selected metabolites in the Random forest model to predict associations between genera and metabolites.

Brand	Genus	Selected metabolites	Adjusted association	Pseudo-*R*^2^
A	*Thermus*	Phe	Positive	0.85
B	*Streptococcus*	Lac	Negative	0.93
		Urea	Negative	
	*Lactococcus*	HepA	Positive	0.98
C	*Streptococcus*	Urea	Negative	0.98
		Cholesterol	Negative	
		Pglu	Negative	
	*Lactococcus*	Urea	Positive	0.96
		Pip	Negative	
		Orn	Negative	0.83
	*Lactobacillus*	Gln	Negative	
		Tyr	Positive	

*Partial plots were used to determine the direction of the associations between the genera and the metabolites (plots are not shown). See Figure 3 for metabolite abbreviations.*

## Discussion

This research demonstrates that similar commercial cheddar cheeses of different maturity levels (ripening ages) made by independent manufacturers can be differentiated by the application of multi-omics-microbiota and metabolomics analyses combined with data integration analysis. Notably, GC-MS untargeted metabolomics identified metabolites that were specific to cheeses of particular ages (maturity) and have the potential for use as markers for monitoring cheese ripening progression, validating cheese age, improving the quality and efficiency of cheese ripening outcomes. The identification of metabolites as diagnostic biomarkers further offers the potential for their inclusion in the cheese grading process. Our research has demonstrated that strong and significant associations exist between cheese microbiota and metabolites. To our knowledge, some of these associations have not been previously described, such as the positive association between the levels of phenylalanine and the presence of *Thermus*, while others were consistent with the known biochemical characteristics of bacterial species, such as the association between urease-positive *S. thermophilus* and decreased levels of urea (Afshari et al., 2020).

*Lactococcus* was the dominant genus in cheeses from brands A and B, presumably reflecting the growth of *Lc. lactis* derived from the cheese starter culture (16S rRNA gene sequence data from amplicons were most closely related to sequences from *Lc. lactis*; data not shown), while *Lactobacillus* was present at ∼16–38% in brand A cheeses and <1% in brand B cheeses. Brand C cheeses were dominated by *Lactobacillus* spp. and *Lactococcus* spp., with the relative abundance of *Lactobacillus* increasing in cheeses of increasing maturity (Figure 2C). This is consistent with the standard cheese ripening model, which predicts that numbers of starter lactococci, which are very high at the start of ripening then decrease, accompanied by growth of *Lactobacillus* spp. derived from added flavor adjunct cultures or other adventitious microflora, or both (Fitzsimons et al., 2001; Stefanovic et al., 2018). In contrast to brands A and C, cheeses from brand B contained very low proportions of *Lactobacillus*, potentially suggesting that *Lactobacillus* were not used as adjunct cultures in the manufacture of brand B cheeses.

*Streptococcus* was present at high relative abundances (up to ∼50%) in both tasty and extra tasty cheeses from brand B, but not in the vintage cheddar. This might be explained if *S. thermophilus* was present in addition to *Lc. lactis* in the starter culture. While not traditionally present in mesophilic (or “O”-type) DVS cheese cultures (Blaya et al., 2018; Høier et al., 2010), some more recent “O”-type cultures do contain added *S. thermophilus* in order to enhance acid production at the cheddar cook stage (see for example Christian Hansen DVS cataloge, 2014, pp. 11–13^4^). Alternatively, though less likely given such high levels in the cheese itself, *S. thermophilus* may have been present as biofilms in the downstream cooling side of the pasteurizer, some of which may have sloughed off into the cheese milk during vat filling (Bouman et al., 1982). Either way, *S. thermophilus* inoculated into the cheese milk during vat filling would increase in numbers even after the cook stage, but would then die off quickly once ripening commenced. In contrast, communities in vintage cheeses from brand B were dominated by *Lactococcus* (Figure 2B); whether a different starter culture that did not contain *S. thermophilus* was used to produce the vintage cheeses (brand B) is not known. This comparison of cheeses from different brands and of differing maturities highlights that both intended or unintended variation in cheese microbiota composition during dairy manufacturing is a variable that needs to be considered and that is likely to influence cheese quality and sensory characteristics, even within cheeses of the same type.

In brand A cheeses, *Thermus* spp. were present at a relative abundance of up to 2.5% but were absent from other brands and their presence may have originated from hot water sources in the factory (Quigley et al., 2016). Based on microbiome DNA sequencing studies, Quigley et al. (2016) suggested that *Thermus* may be the causative agent of pink discoloration in cheese, a problem that has affected the dairy industry over many years (Daly et al., 2012) and yet remains without a definitive explanation. In our study, the presence of this genus was associated with high levels of phenylalanine (Table 1), although none of the cheeses in our study exhibited a pink discoloration defect. This is consistent with the observations of Quigley et al. (2016) who found no association between the presence of free amino acids and the development of pink discoloration. Rather, these authors have suggested that the defect may be due to a microbially produced carotenoid when *Thermus* is present at a higher relative abundance (up to 6% of the total 16S rRNA reads).

Cheese is known to contain many thousands of metabolites present in varying abundance (Afshari et al., 2020). Although the impact of metabolites found in low relative abundances remains largely unexplored, there is evidence that some may have significant impacts on cheese flavor and quality. For example, esters present in very low amounts in cheese can be detected in taste testing (Holland et al., 2005). GC-MS untargeted profiling revealed differences in metabolite profiles between the three different brands of cheddar as well as between cheeses of different maturity and additionally, identified cheese metabolites that were correlated with aging. These results are in agreement with other research that suggests metabolome fingerprinting may be a useful indicator of cheese maturity (Ochi et al., 2013; Gan et al., 2016). As cheddar cheeses mature, proteolysis and lipolysis results in release of peptides, amino acids, and free fatty acids, all contributing to flavor (McSweeney, 2004). Our research has shown progressive increases in the production of seven carboxylic acids together with 16 amino acids and one heterocyclic amine (piperidine) during cheese ripening in cheeses from brand A and C cheeses. This is consistent with previous studies that reported an increase in amino acids, especially lysine, proline, glycine and pyroglutamic acid with ripening time (Ardo et al., 2002; Zheng et al., 2018). The association of threonine with aged cheddars (30 months ripening) and of isoleucine and leucine with relatively younger (24 months ripening) hard cheeses has also been reported (Consonni and Cagliani, 2008). Mucchetti et al. (2000) also reported a linear correlation between the concentration of pyroglutamic acid and ripening age with the age of extensively ripened Italian Grana Padano cheese. Furthermore, in contrast to brands A and C, the less mature tasty cheeses from brand B were characterized by a higher relative abundance of amino acids than in the more mature extra tasty and vintage cheeses (Figure 3C). Such differences in amino acid abundance between brands could be due to differences in processing methods such as salt content, geography and the microbial compositions of different cheeses made by different manufacturers (Yvon and Rijnen, 2001; Masotti et al., 2010; Moser et al., 2018).

Cheese is a complex ecosystem where many metabolites can be re-metabolized or catabolized by multiple microbial species (Irlinger and Mounier, 2009). In addition, enzymes released into the curd even after cell death may continue to catalyze reactions. This means that relationships between cheese microbiota and metabolites are unlikely to be linear. A model which allows many different types of relationships, including complex interactions, is expected to be more accurate and versatile in predicting associations between microbiota and metabolites. Hence, in this study, the RF regression model (Breiman, 2001) was used to predict such associations between cheese microbiota and cheese metabolites. Similarly, MFA integrative analysis showed that the overall (global) associations between microbiota and metabolite composition in cheeses varied between brands. The higher associations seen between bacterial taxa and metabolites for brand C (RV = 0.73) as revealed by MFA analysis, may be due to its different microbial community structure and/or varying succession, since different microbes will possess different enzymatic capacities affecting metabolite production. It is to be noted that DNA sequencing of PCR amplified 16S rRNA genes (as has been performed in this study) will detect both live and dead cells (see Emerson et al., 2017) and not specifically identify those cells that are active. There is a complex interplay between growth of specific genera and subsequent death and lysis of cells, and the subsequent production of metabolites in cheese, since many enzymes such as peptidases released by bacterial autolysis remain active after cell death. Indeed, some enzymes appear to be more stable in the cheese environment than they are in intact stressed cells (Crow et al., 1995). This makes validation of biomarkers using more diverse and larger sample sets extremely important. Furthermore, the RF regression modeling for brand C cheeses showed that both cholesterol and urea were negatively associated with the abundance of *Streptococcus* (*S. thermophilus*). The ability of several strains of LAB, including *Streptococcus thermophilus* (and also *Lactobacillus*) species to reduce cholesterol levels in vitro and in cheese matrix has previously been demonstrated (Albano et al., 2018; Belviso et al., 2009; Ziarno et al., 2007). Understanding of this association between certain LAB and reduced cholesterol levels offers opportunities to improve human health in relation to cheese consumption.

In terms of other functional relationships between microbiota and metabolites, the presence and abundance of *Lactococcus* was associated with decreased levels of amino acids in brand C cheeses (as shown by MFA and RF) and in particular, with pyroglutamic acid and piperidine (a heterocyclic amine) both of which have previously been reported in mature cheeses (Golovnya et al., 1969). It has long been established that *Lactococcus lactis* contributes to cheese flavor by metabolizing amino acids and converting them into flavorsome compounds, for example, by deamination to α-ketoacids and subsequent conversion to amino acids to aldehydes, esters, alcohols and carboxylic acids (Kieronczyk et al., 2003). However, the biochemistry which underlies the negative association between piperidine and *Lactococcus* warrants further investigation. The contribution of this amine to cheese flavor is unknown. The RF optimized model also showed that the relative abundance of *Lactobacillus* was inversely correlated with ornithine and glutamine in brand C cheeses. Ornithine, produced by the decarboxylation activity of LAB through the arginine deiminase (ADI) pathway, has been shown to be physiologically active (Kurata et al., 2011; Zúñiga et al., 2002). The ability of *Lactobacillus paracasei* to convert a wide range of amino acids including glutamine and ornithine, but not pyroglutamic acid *in vitro* has been shown previously (Tammam et al., 2000). Similarly, for brand B cheeses we determined the overall correlation of 57% (RV = 0.57) between microbiota and metabolites composition by MFA. Some of these correlations between microbiota and metabolites were of interest; for example, a positive association between a low -abundant taxa, *Acinetobacter* (a common spoilage organism) (<0.1% of total reads) and medium chain fatty acids. This association may be due to the ability of this genus to produce lipase in the milk and/or cheese (Pratuangdejkul and Dharmsthiti, 2000). However, the effects of lipolysis on milk quality cannot be discounted (Hickey et al., 2007).

While our findings cannot prove causation, they demonstrate that the metabolome profiles of cheeses (and of individual metabolites therein) which influence cheese quality and flavor also may be a useful predictor of the microbial composition of cheeses (Gallegos et al., 2017). This improved understanding could additionally be applied to informing decision-making on choice of “desirable” starter or adjunct cultures to optimize cheese quality and flavor. Future targeted and controlled studies involving more diverse and larger sample sets and whole genome sequencing for differentiation of species and strains, together with detailed profiling of volatile and non-volatile metabolites and sensory analysis, are needed to validate the associations between cheese microbiota and metabolomes and identify potential biomarkers for monitoring cheese quality and authenticity.

## Data Availability Statement

The original contributions presented in the study are publicly available. This data can be found here in NCBI, under accession PRJNA673975.

## Author Contributions

RA designed and performed the experiments, analyzed the data, and drafted the manuscript. Other authors listed made a substantial, direct and intellectual contribution to the work, and approved the manuscript for publication.

## Conflict of Interest

The authors declare that the research was conducted in the absence of any commercial or financial relationships that could be construed as a potential conflict of interest.

## Footnotes

1 http://www.nist.gov

2 http://csbdb.mpimp-golm.mpg.de/csbdb/dbma/msri.html

3 http://factominer.free.fr/

4 Available at https://hjemmeriet.com/da/ChrHansen/Brochures/GlobalCheese CultureCatalogue_EN-2014.pdf.
