# Supplementary material for: Microbiota and Metabolite Profiling Combined With Integrative Analysis for Differentiating Cheeses of Varying Ripening Ages
Source: Front Microbiol. 2020 Nov 26;11:592060. doi: 10.3389/fmicb.2020.592060 (PMC7726019; doi:10.3389/fmicb.2020.592060)
Supplement: Supplementary file 1 [file Image_1.pdf]

Supplementary Material

A Variable importance for *Thermus* (brand A)

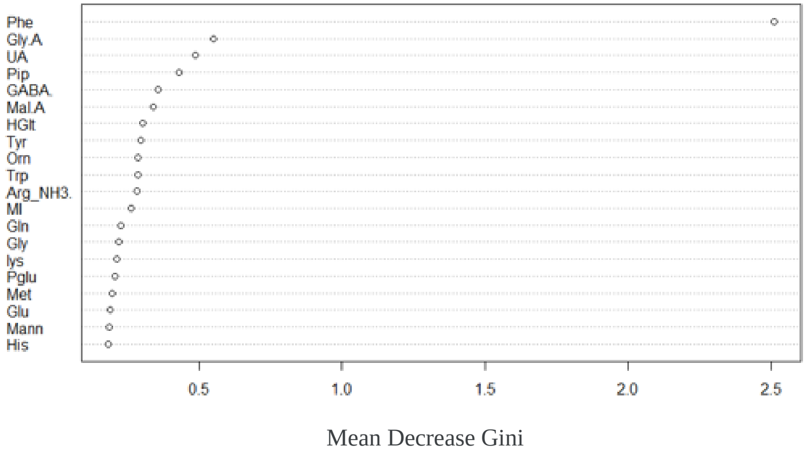

B Variable importance for *Streptococcus* (brand B)

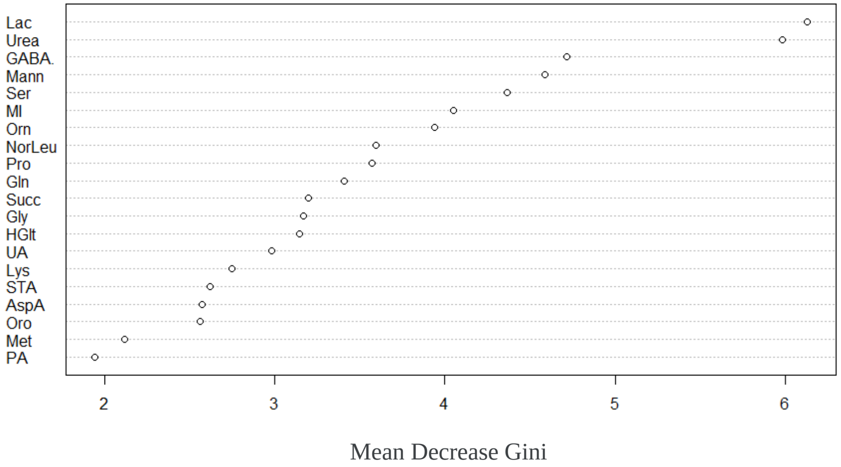

C Variable importance for *Lactococcus* (brand B)

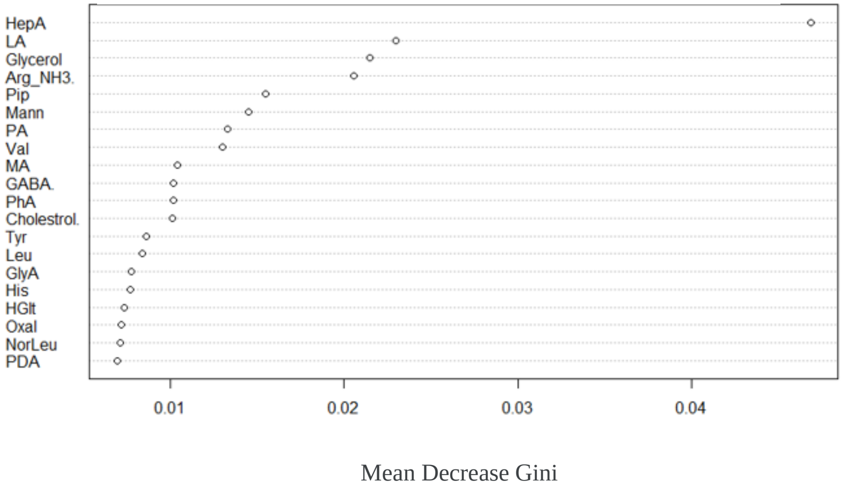

D Variable importance for *Streptococcus* (brand C)

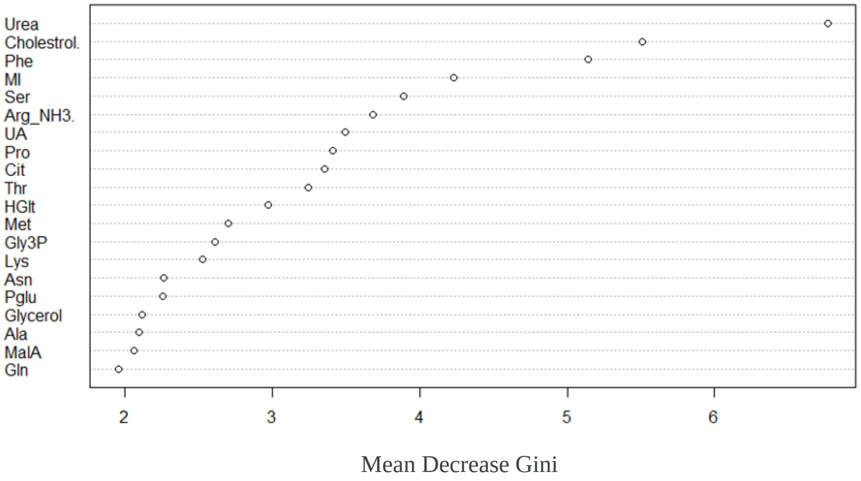

E

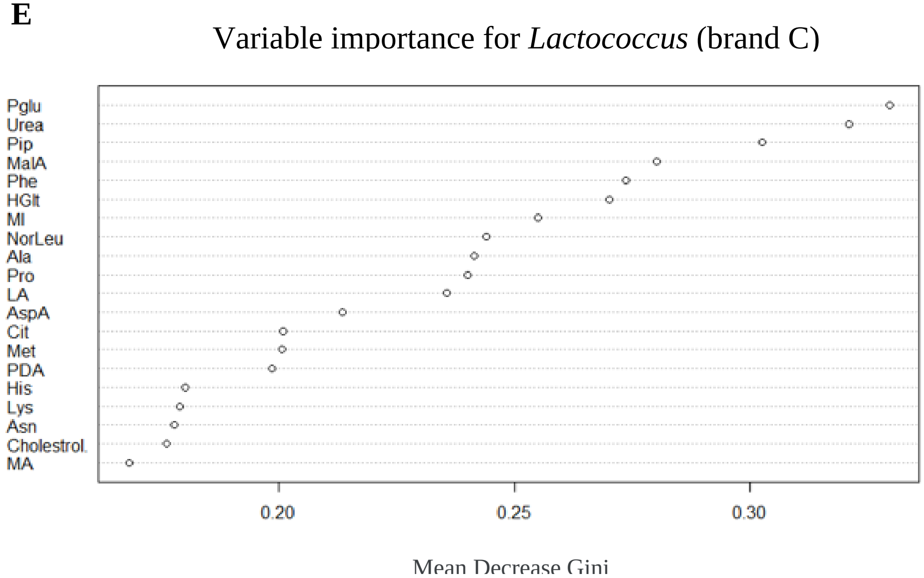

F

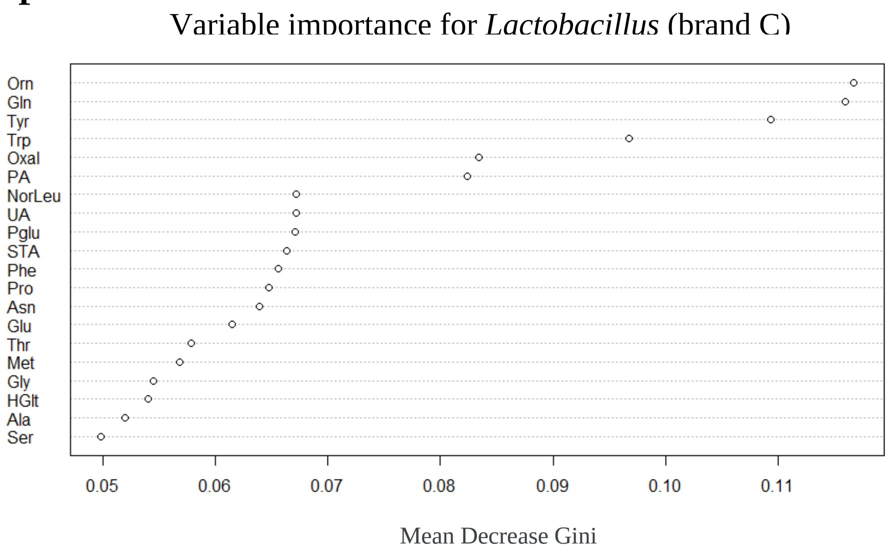

Figure S1. Random forest regression model to predict the association between cheese bacterial genera and metabolites. Variable importance plots showed which metabolites (response data) in the random forests optimised models had the highest association with the relative abundance of: **(A)** *Thermus* (brand A), **(B)** *Streptococcus* (brand B), **(C)** *Lactococcus* (brand B), **(D)** *Streptococcus* (brand C), **(E)** *Lactococcus* (brand C) and **(F)** *Lactobacillus* (brand C). The higher the Mean Gini value the more important the variable in the optimized model.
