# Supplementary material for: Microbiota and Metabolite Profiling Combined With Integrative Analysis for Differentiating Cheeses of Varying Ripening Ages
Source: Front Microbiol. 2020 Nov 26;11:592060. doi: 10.3389/fmicb.2020.592060 (PMC7726019; doi:10.3389/fmicb.2020.592060)
Supplement: Supplementary file 2 [file Table_1.pdf]

Supplementary Material

Table S1. The GC-MS characteristics of cheese metabolites

Compound	Abbreviation	Retention time (min)	EI-MS unique fragment ion
Alanine (2TMS[1])	Ala	7.78	190
Glycine (2TMS)	Gly	8.1	147
Valine (2TMS)	Val	9.41	218
Urea (2TMS)	Urea	10.47	189
Phosphoric acid (3TMS)	PhA	10.56	299
leucine (2TMS)	Leu	10.49	158
Norleucine	NorLeu	10.9	158
Proline (2TMS)	Pro	11.11	142
Succinic acid (2TMS)	Succ	11.42	247
Glyceric acid (3 TMS)	GlyA	11.61	292
Uracil (2TMS)	Uracil	11.98	255
Serine (2TMS)	Ser	10.37	219
Threonine (2TMS)	Thr	11.04	248
Piperidine (2TMS)	Pip	13.96	258
Aspartic acid (2TMS)	AspA	15.03	232
Pyroglutamic acid (2TMS)	PGlt	15.15	258
γ-Aminobutyric acid (3TMS)	GABA	15.2	304
Phenylalanine (1TMS)	Phe	16.98	220
2-Hydroxyglutaric acid (3TMS)	HGlt	16.07	349
Ornithine (3TMS)	Orn	16.63	348
Glutamic acid (2TMS)	Glu	16.18	246
Asparagine (3TMS)	Asn	17.65	231
Lysine (3TMS)	lys	18.13	230
Glutamine (3TMS)	Gln	15.53	155
Mannose (5TMS, 1 MeOX)[1]	Mann	20.93	160
Tyrosine (2TMS)	Tyr	20.99	218
Histidine (3TMS)	His	21.43	254
Palmitic acid (ITMS)	PA	23.162	313
Tryptophan (2TMS)	Trp	25.36	202
Stearic acid (1TMS)	STA	25.69	341
Lactose, (8TMS, 1 MEOX)	Lac	30.595	480
Cholesterol (1TMS)	Cholesterol	35.41	458
Oxalic acid	Oxal	8.54	190
Malonic acid (2TMS)	MalA	9.18	245
Glycerol (3TMS)	Glycerol	10.58	205
Methionine (1TMS)	Met	13.33	221
Lauric acid (1TMS)	LA	17.73	257
Ornithine (3TMS)	Oro	18.74	254
Glycerin 3phosphate (3TMS)	Gly3P	19.07	299

Citric acid (4TMS)	Cit	19.93	465
Arginine-NH3 (3TMS)	Arg-NH3	19.96	256
Myristic acid (1TMS)	MA	20.38	285
Galactose	Gal	21.262	160
Pentadecanoic acid, n-(1TMS)	PDA	21.92	401
Inositol-myo (6TMS)	MI	23.68	305
Uric acid (4TMS)	UA	23.79	441
Heptadecanoic acid, n-(1TMS)	HepA	24.55	327

TMS = trimethylsilyl derivate; MEOX = methyloxime derivate.
